# Resting-state functional connectivity in anxiety disorders: a multicenter fMRI study

**DOI:** 10.1038/s41380-024-02768-2

**Published:** 2024-10-04

**Authors:** Till Langhammer, Kevin Hilbert, Dirk Adolph, Volker Arolt, Sophie Bischoff, Joscha Böhnlein, Jan C. Cwik, Udo Dannlowski, Jürgen Deckert, Katharina Domschke, Ricarda Evens, Thomas Fydrich, Bettina Gathmann, Alfons O. Hamm, Ingmar Heinig, Martin J. Herrmann, Maike Hollandt, Markus Junghoefer, Tilo Kircher, Katja Koelkebeck, Elisabeth J. Leehr, Martin Lotze, Jürgen Margraf, Jennifer L. M. Mumm, Andre Pittig, Jens Plag, Jan Richter, Kati Roesmann, Isabelle C. Ridderbusch, Silvia Schneider, Hanna Schwarzmeier, Fabian Seeger, Niklas Siminski, Thomas Straube, Andreas Ströhle, Christoph Szeska, Hans-Ulrich Wittchen, Adrian Wroblewski, Yunbo Yang, Benjamin Straube, Ulrike Lueken

**Affiliations:** 1 Department of Psychology, Humboldt-Universität zu Berlin, Berlin, Germany; 2 Department of Psychology, HMU Health and Medical University Erfurt, Erfurt, Germany; 3 Mental Health Research and Treatment Center, Faculty of Psychology, Ruhr-Universität Bochum, Bochum, Germany; 4 Institute for Translational Psychiatry, University of Münster, Münster, Germany; 5 Department of Psychiatry and Psychotherapy, Campus Mitte, Charité-Universitätsmedizin Berlin, Berlin, Germany; 6 Department of Clinical Psychology and Psychotherapy, University of Cologne, Cologne, Germany; 7 Department of Psychology and Psychotherapy, Witten/Herdecke University, Witten, Germany; 8 Department of Psychiatry, Psychosomatics and Psychotherapy, Center of Mental Health, University of Würzburg, Würzburg, Germany; 9 Department of Psychiatry and Psychotherapy, Medical Center—University of Freiburg, Faculty of Medicine, University of Freiburg, Freiburg im Breisgau, Germany; 10 Institute of Medical Psychology and Systems Neuroscience, University of Münster, Münster, Germany; 11 Department of Biological and Clinical Psychology, University of Greifswald, Greifswald, Germany; 12 Institute for Clinical Psychology and Psychotherapy, Technical University of Dresden, Dresden, Germany; 13 Department of Clinical Psychology and Psychotherapy, University of Greifswald, Greifswald, Germany; 14 Institute for Biomagnetism and Biosignalanalysis, University of Münster, Münster, Germany; 15 Otto Creutzfeldt Center for Cognitive and Behavioral Neuroscience, University of Münster, Münster, Germany; 16 Department of Psychiatry and Psychotherapy and Center for Mind, Brain and Behavior - CMBB, Philipps-Universität Marburg, Marburg, Germany; 17 LVR-University Hospital Essen, Department of Psychiatry and Psychotherapy, University of Duisburg-Essen, Duisburg, Germany; 18 Functional Imaging Unit, Diagnostic Radiology, University Medicine Greifswald, Greifswald, Germany; 19 Translational Psychotherapy, Institute of Psychology, University of Goettingen, Göttingen, Germany; 20 Department of Medicine, Institute for Mental Health and Behavioral Medicine, HMU Health and Medical University, Potsdam, Germany; 21 Department of Experimental Psychopathology, University of Hildesheim, Hildesheim, Germany; 22 Institute for Psychology, Clinical Psychology and Psychotherapy in Childhood and Adolescence, University of Osnabrueck, Osnabruck, Germany; 23 Department of Clinical Child and Adolescent Psychology, Ruhr-University Bochum, Bochum, Germany; 24 Center for Mental Health, Department of Psychiatry, Psychosomatics, and Psychotherapy, University Hospital of Würzburg, Würzburg, Germany; 25 Department of Biological Psychology and Affective Science, University of Potsdam, Potsdam, Germany; 26 Department of Psychiatry and Psychotherapy, Ludwig-Maximilians-University (LMU) Muenchen, Munich, Germany; 27 German Center for Mental Health (DZPG), partner site Berlin/Potsdam, Berlin, Germany

**Keywords:** Diagnostic markers, Neuroscience, Psychology, Psychiatric disorders, Predictive markers

## Abstract

Anxiety disorders (AD) are associated with altered connectivity in large-scale intrinsic brain networks. It remains uncertain how much these signatures overlap across different phenotypes due to a lack of well-powered cross-disorder comparisons. We used resting-state functional magnetic resonance imaging (rsfMRI) to investigate differences in functional connectivity (FC) in a cross-disorder sample of AD patients and healthy controls (HC). Before treatment, 439 patients from two German multicenter clinical trials at eight different sites fulfilling a primary diagnosis of panic disorder and/or agoraphobia (PD/AG, N = 154), social anxiety disorder (SAD, N = 95), or specific phobia (SP, N = 190) and 105 HC underwent an 8 min rsfMRI assessment. We performed categorical and dimensional regions of interest (ROI)-to-ROI analyses focusing on connectivity between regions of the defensive system and prefrontal regulation areas. AD patients showed increased connectivity between the insula and the thalamus compared to controls. This was mainly driven by PD/AG patients who showed increased (insula/hippocampus/amygdala—thalamus) and decreased (dorsomedial prefrontal cortex/periaqueductal gray—anterior cingulate cortex) positive connectivity between subcortical and cortical areas. In contrast, SAD patients showed decreased negative connectivity exclusively in cortical areas (insula—orbitofrontal cortex), whereas no differences were found in SP patients. State anxiety associated with the scanner environment did not explain the FC between these regions. Only PD/AG patients showed pronounced connectivity changes along a widespread subcortical-cortical network, including the midbrain. Dimensional analyses yielded no significant results. The results highlighting categorical differences between ADs at a systems neuroscience level are discussed within the context of personalized neuroscience-informed treatments. PROTECT-AD’s registration at NIMH Protocol Registration System: 01EE1402A and German Register of Clinical Studies: DRKS00008743. SpiderVR’s registration at ClinicalTrials.gov: NCT03208400.

## Introduction

Anxiety disorders (AD), including panic disorder and/or agoraphobia (PD/AG), social anxiety disorder (SAD), and specific phobia (SP), are among the most common mental disorders. Their 12-month prevalence lies between 14.0% (EU rates; [1]) and 18.1% (US rates; [2]), posing a substantial challenge to both patients and society [3]. Modern psychopathological models advocate for a transdiagnostic viewpoint, emphasizing underlying similarities that transcend diagnostic labels [4]. Notably, ADs have common developmental patterns, risk factors, symptoms, and treatment strategies, with exposure-based cognitive behavioral therapy recommended as the primary approach. However, the underlying nature of the commonalities and differences, including phenotypic homo- or heterogeneity of neural signatures, is still not well-understood [5].

ADs are characterized by dysfunctional threat-processing mechanisms that involve specific brain regions. Pre-clinical research, including animal studies, has consistently identified key areas within the defensive system, with high translational value for understanding the neurobiological basis of anxiety disorders [6, 7]. Meta-analyses using multimodal neuroimaging highlight common neural features in internalizing disorders, especially ADs [8–10]. A meta-analysis with over 15,000 participants identified shared structural alterations in the dorsal ACC and bilateral insula [8]. Further, heightened activity is observed in the salience network, particularly the anterior cingulate cortex (ACC) and insula, alongside decreased activity in prefrontal and executive control regions. Task-based fMRI studies have shown increased activation in anxiety-related areas, such as the bilateral insula and medial prefrontal cortex [10]. Common neural patterns for mood and anxiety disorders include reduced activity in frontal areas that exert inhibitory functions and increased activity in salience-processing networks (amygdala, ACC, and thalamus; [8]). However, these meta-analyses predominantly emphasize shared neural characteristics, often overlooking distinctions between diagnoses.

Resting-state fMRI offers a paradigm-free measure of intrinsic brain connectivity, positioning it as a temporally stable characteristic of individuals and potential biomarker [11]. Yet, studies on resting-state functional connectivity (rsFC) as a function of diagnosis and/or psychopathology related to AD are limited. The most striking results show connectivity changes between limbic and associated regions (e.g., amygdala and insula) as well as regions associated with the default mode network, salience network, and central executive network [5, 11–13]. Findings are, however, heterogenous, possibly due to different MRI techniques and analysis methods. Many studies also lack statistical power [5]. Despite indications of both shared and distinct rsFC patterns among ADs, comprehensive and direct cross-disorder comparisons are rare, making the true nature of shared neural markers in transdiagnostic models debatable.

We aimed to analyze rsFC variations between different ADs and healthy controls (HC) using a direct cross-disorder comparison on a sample covering PD/AG, SAD, and SP, sourced from two major multicenter studies (PROTECT-AD and SpiderVR). Based on previous studies [8–13], we hypothesized variations in connectivity within the defense mobilization network (comprising regions from both the executive control and salience networks, such as the medial prefrontal cortex, amygdala, ACC, insula, and thalamus), including the periaqueductal gray (PAG), and prefrontal regulatory regions. We explored differences between AD patients and HC in general, as well as between specific diagnoses (in the Supplemental Material).

## Methods

The analysis is part of the national research consortium “Providing Tools for Effective Care and Treatment of Anxiety Disorders” (PROTECT-AD), sponsored by the German Federal Ministry of Education and Research. Patients with PD/AG, SAD, or SP diagnoses from eight German university outpatient clinics participated (details in [14, 15]). At baseline, 468 patients and controls underwent rsfMRI. Due to limited SP representation, we added 174 SP (spider phobia) patients from the SpiderVR trial, funded by the German Research Foundation within the “Fear, Anxiety, Anxiety Disorders” Collaborative Research Center (CRC TRR 58, project C9; details in [16, 17]). This trial used the same neuroimaging protocols, including rsfMRI, at two sites also involved in PROTECT-AD.

### Participants

Data sets of 439 AD patients and 105 HC patients after quality control were included (see Supplemental Material for details). PROTECT-AD patient eligibility was based on the Diagnostic and Statistical Manual of Mental Disorders (DSM 5th edition) criteria for primary diagnoses of PD, AG, SAD, or multiple SP. For SpiderVR, eligibility hinged on the DSM (IV-TR) criteria for spider phobia. HC participants had no history of mental illness or a neurological or medical condition preventing MRI.

**Fig. 1:**
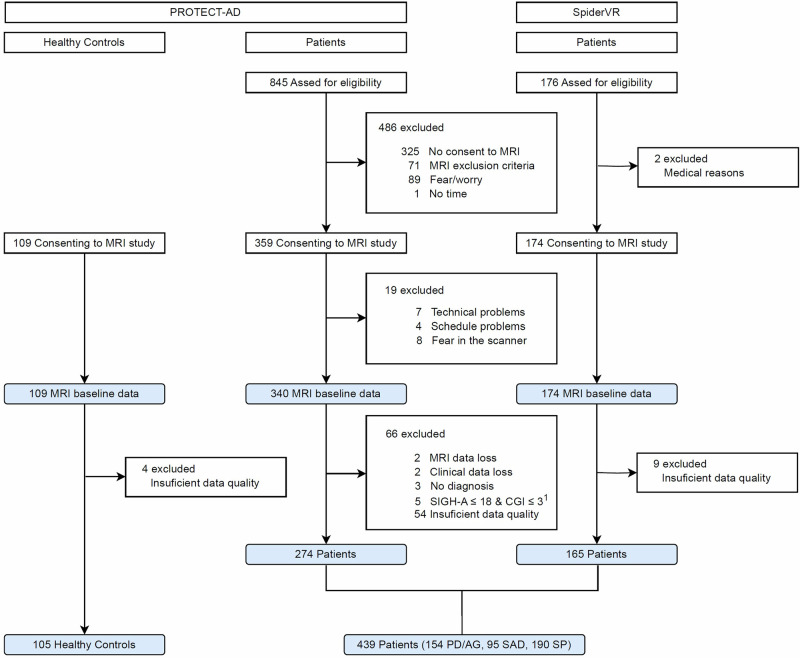
Participant flowchart. ^1^for pilot patients (n = 31) in PROTECT-AD inclusion criteria were different with either SIGH-A or CGI being above cut-off. For details see Supplemental Material.

Participants provided written consent after receiving a full description of the study protocol. All ethics committees of the participating centers approved the study. A patient flow diagram is available in Fig. 1, while demographic and clinical data can be found in Table 1.

**Table 1:** Clinical and demographic characteristics (means and SDs except otherwise specified).

Characteristics	All patients (N = 439)		PD/AG (N = 154)		SAD (N = 95)		SP (N = 190)		HC (N = 105)		*χ*^*2*^/*F* (df1,df2)		*p*
Female gender, n (%)	289	(65.83)	81	(52.60)	51	(53.68)	157	(82.63)	54	(51.43)	48.16	(3)	<0.001^c^
Age (years)	29.70	(9.76)	32.16	(10.44)	27.16	(6.12)	28.98	(10.00)	31.96	(10.04)	7.39	(3,540)	<0.001^d^
Years of Education	12.97	(2.55)	11.69	(1.42)	12.11	(1.21)	14.36	(2.97)	12.25	(1.38)	54.20	(3)	<0.001^e^
Living alone, n (%)	168	(38.27)	70	(45.45)	39	(41.05)	59	(31.05)					
Smoking, n (%)	72	(16.40)	41	(26.62)	22	(23.16)	9	(4.74)	20	(19.05)	22.90	(2)	<0.001^f^
Left handed, n (%)	28	(6.38)	15	(9.74)	12	(12.63)	1	(0.05)				(2)	<0.001^g^
Disease duration (years)	17.69	(13.72)	13.51	(11.82)	15.17	(12.72)	22.34	(14.28)			21.52	(2)	<0.001^h^
Number of diagnoses, n (%)	1.90	(1.12)	2.71	(1.82)	2.0	(0.93)	1.2	(0.57)			118.38	(2)	<0.001^i^
Current stable medication, n (%)													
• None	368	(83.83)	111	(72.08)	69	(72.63)	188	(98.95)			56.50	(2)	<0.001^j^
• Painkillers	11	(2.50)	4	(2.60)	5	(5.26)	2	(1.05)			4.60	(2)	0.100
• Sleep-inducing agents	3	(0.07)	3	(1.95)	0	(0.00)	0	(0.00)			1.87	(2)	0.394
• Tranquilizers	6	(1.37)	5	(3.25)	1	(1.05)	0	(0.00)			6.74	(2)	0.034
• Stimulants	0	(0.00)	0	(0.00)	0	(0.00)	0	(0.00)			–	–	–
• Antidepressants	60	(13.67)	39	(25.32)	21	(22.34)	0	(0.00)			53.55	(2)	<0.001^k^
• Mood stabilizers	2	(0.00)	0	(0.00)	2	(2.11)	0	(0.00)			7.28	(2)	0.026
• Neuroleptics	3	(0.00)	1	(0.65)	2	(2.11)	0	(0.00)			4.14	(2)	0.126
Symptom severity													
• SIGH-A total^a^	24.38	(5.94)	24.73	(5.94)	24.28	(6.08)	22.56	(5.17)			1.46	(2,271)	0.235
• CGI	4.69	(0.76)	4.93	(0.70)	4.95	(0.67)	4.37	(0.74)			34.52	(2,436)	<0.001^l^
• PAS	18.31	(10.40)	22.06	(9.26)	13.83	(9.72)	12.52	(10.46)			26.83	(2,269)	<0.001^m^
• LSAS	49.25	(32.40)	37.66	(25.03)	72.28	(25.56)	47.12	(34.93)			40.76	(2,435)	<0.001^n^
• DSM-5 SP^b^	12.74	(9.74)	14.25	(9.68)	10.46	(9.34)	12.65	(9.79)			11.12	(2,271)	<0.001^o^
• ASI	22.30	(12.50)	29.04	(10.88)	24.19	(9.47)	15.89	(11.90)	8.67	(6.20)	61.87	(2,436)	<0.001^p^
• BDI-II	11.67	(10.18)	15.71	(9.00)	19.16	(10.06)	4.62	(5.71)	2.28	(3.37)	133.62	(2,435)	<0.001^q^
Site/scanner, n (%)													
• TrioTrim 1	60	(13.7)	29	(18.8)	30	(31.6)	1	(0.5)	15	(14.3)			
• Verio	19	(4.3)	7	(4.5)	6	(6.3)	6	(3.2)	10	(9.5)			
• TrimTrio 2	59	(13.4)	31	(20.1)	24	(25.3)	4	(2.1)	14	(13.3)			
• Skyra (SpiderVR & PROTECT-AD)	112	(25.5)	14	(9.1)	10	(10.5)	88	(46.3)	15	(14.3)			
• Philipps	28	(6.4)	16	(10.4)	11	(11.6)	1	(0.5)	14	(13.3)			
• TrimTrio 3 (shared by two sites)	56	(12.8)	42	(27.3)	9	(9.5)	5	(2.6)	26	(34.8)			
• Prisma (SpiderVR & PROTECT-AD)	105	(23.9)	15	(9.7)	5	(5.3)	85	(44.7)	11	(10.5)			

All patients N = 439; PD/AG N = 154; SAD N = 95; SP N = 190

*SIGH‐A* structured interview guide for the Hamilton anxiety rating scale, *CGI* clinical global impression scale, *BSI* brief symptom inventory, *BDI‐II* beck depression inventory‐II, *ASI* anxiety sensitivity index, *PAS* panic and agoraphobia scale, *LSAS* Liebowitz social anxiety scale, *DSM‐5 SP Scale* dimensional specific phobia scale for DSM‐5.

^a^Only patients from PROTECT-AD (N = 273).

^b^Only SP patients from PROTECT-AD (N = 25).

^c^HC < SP

^d^SAD < HC

^e^PD/AG, SAD, HC < SP

^f^SP < SAD, HC < PD/AG

^g^SP < PD/AG, SAD

^h^PD/AG, SAD < SP

^i^PD/AG, SAD > SP

^j^PD/AG, SAD > SP

^k^SP < SAD < PD/AG

^l^SP < PD/AG, SAD

^m^SAD, SP < PD/AG

^n^PD/AG < SP < SAD

^o^SAD < SP < PD/AG

^p^HC < SP < PD/AG, SAD

^q^HC < SP < PD/AG, SAD

### Clinical assessments

In PROTECT-AD, diagnoses were made by trained clinicians using a standardized computerized interview based on DSM-5 criteria [16]. For SpiderVR, the DSM-IV structured clinical interview was employed [17]. Differences between DSM-5 and DSM-IV did not affect decisions regarding specific phobia diagnoses. Selected measures for analysis included: Structured Interview Guide for the Hamilton Anxiety Rating Scale (SIGH-A; [18]), Clinical Global Impression Scale (CGI; [19]), Panic and Agoraphobia Scale (PAS; [20]), Liebowitz Social Anxiety Scale (LSAS; [21]), Dimensional Specific Phobia Scale for DSM-5 (DSM5-SP; [22]), Anxiety Sensitivity Index (ASI-3; [23]), Beck Depression Inventory (BDI-II; [24]), and Spider Phobia Questionnaire (SPQ; [25]).

PROTECT-AD participants completed an additional questionnaire assessing the course of anxiety during scanning and maximal anxiety to test the state anxiety associated with the procedure (see [26] for more details).

### MRI Acquisition

MRI scans were performed at eight clinical sites using seven 3 Tesla MRI scanners (3x Siemens TrioTim, 1x Siemens Verio, 1x Siemens Prisma, 1x Siemens Skyra, 1x Philips Achieva). Two sites shared one scanner. SpiderVR and PROTECT-AD used identical scanner sequences. Data quality assurance was achieved through harmonized scanner sequences, trained personnel, frequent site visits, teleconferences, and rapid online data quality checks with direct feedback to each center (see Supplemental Material for more information).

Functional images at rest were obtained after the structural images and before the task-based images in both trials. This was done using an 8-minute T2-weighted gradient-echo echo-planar imaging (EPI) sequence, which is sensitive to BOLD contrast (TE = 30 ms, TR = 2000 ms, flip angle 90°, matrix size 64 × 64 voxels, voxel size 3.3 × 3.3 × 3.8 mm³, slice thickness 3. 8 mm, slice spacing 0.38 mm, field of view (FOV) = 210 mm, 33 slices scanned in order interleaved ascending with phase encoding direction A ≫ P; due to technical incompatibility, a TE = 29 ms had to be used on the Siemens Prisma, only 31 slices were acquired on the Siemens Verio). The slices were positioned transaxially parallel to the intercommissural (AC-PC) plane and tilted 20° to reduce magnetic susceptibility artifacts in prefrontal areas. Participants were instructed to remain still and close their eyes. The light in the MRI scanning room was turned off.

A high-resolution structural image was obtained using a three-dimensional T1-weighted magnetization-prepared fast gradient echo sequence (3D MPRAGE) in the sagittal plane (TE = 2.26 ms, TR = 1900 ms, inversion time (TI) = 900 ms, flip angle 9°, matrix size 256 × 256 voxels, voxel size 1 × 1 × 1 mm, slice thickness 1.0 mm, FOV = 256 mm, 176 slices; due to technical incompatibility, a TE = 2.28 ms, TR = 2130 ms, and flip angle = 8° had to be used on the Siemens Prisma; total duration: 4:30 min). Additional fMRI tasks were performed that are not reported here (see [16] and [14] for details of the study protocol). A fixed order was followed (T1, resting state, tasks).

### Data Preprocessing

MRI data were preprocessed using CONN functional connectivity toolbox 19.b (http://www.nitrc.org/projects/conn) implemented in MATLAB, R2019b (MathWorks Inc. MATLAB. Natick, Massachusetts) and SPM12 (Statistical parametric mapping: the analysis of functional brain images. Elsevier). The first five scans of the total 237 volumes were removed from the data before preprocessing. Standard preprocessing steps included realignment/motion correction, slice timing, identification of outlier volumes (≥0.5 mm motion or ≥3 standard deviations of global signal change), direct segmentation and normalization to the standard Montreal Neurological Institute (MNI) brain template, and spatial smoothing (full width at half maximum = 8 mm). Denoising was performed using temporal bandpass filtering (0.008–0.09 Hz) and ordinary least squares (OLS) regression to project each BOLD signal time series onto the subspace orthogonal to all potentially interfering effects. An anatomical component-based noise correction procedure was implemented to identify spurious effects (aCompCor; [27]). Factors identified as confounding effects for the BOLD signal were estimated and removed separately for each voxel and for each participant (see Supplemental Material for further details).

### Data analysis

Sample characteristics were analyzed using χ^2^-tests for differences between groups for dichotomous variables and one-way analyses of variances (ANOVA) for continuous variables. We used multiple χ^2^-tests and Tukey’s HSD Tests for post hoc comparison of single groups. We performed all analyses in IBM SPSS Statistics 26 (significance level of 0.05).

We analyzed the rsFC of a priori selected brain regions in a ROI-to-ROI approach. ROIs were implemented in CONN using the brainnetome atlas [28] and the atlas of the basal ganglia (ATAG; [29]). Based on previous findings [8, 10, 30–32], we selected the PAG, amygdala, ACC (divided into dorsal, pregenual, and subgenual ACC), insula (divided into posterior and anterior insula), hippocampus, thalamus, and prefrontal cortex (divided into dorsomedial PFC [dmPFC], dorsolateral PFC [dlPFC], ventromedial PFC [vmPFC], ventrolateral PFC [vlPFC], and orbitofrontal cortex [OFC]) as ROIs. Regions were recorded separately for both hemispheres unilaterally. Details of the ROI definition are provided in the Supplemental Material.

ROI-to-ROI analyses were performed at the individual level in the CONN toolbox. Temporal correlations of BOLD signals were calculated for all 378 pairwise ROI combinations (28 ROIs). Functional connectivity values were calculated at the group level by calculating bivariate correlations between ROIs (and then converted to Fishers Z correlations).

First, we ran two second-level categorical models: one with patient groups with a primary diagnosis, another with patient groups regardless of whether the diagnosis was a primary or a comorbid diagnosis. We compared all patients with HC, and PD/AG, SAD, and SP separately with HC. For direct comparisons between patient groups, see Supplemental Material. One-way analyses of covariance were performed. Age, sex, and scanner were entered as covariates of no interest.

Second, we examined the dimensional effect of symptom severity (SIGH-A, CGI) on rsFC of all 378 ROI pairs using multiple regression analyses on fixed-level other effects (sex, age, and scanner) within the combined patient group and for individual AD using the PAS, LSAS, and DSM-5 SP. Following the defensive system rationale that differentiates between threat and fear, we additionally examined the associations between the panic subscale and agoraphobic avoidance subscales and connectivity in PD/AG patients [33].

Third, in a post hoc analysis, we examined the effect of maximum anxiety levels during scanning for ROIs that were found to be significant in the categorical model (primary disorder) using multiple regression analysis (effect of state anxiety at fixed values for age, sex, and scanner).

The FDR algorithm of Benjamini & Hochberg [34] was used to control for family-wise error rates, and a connection-level FDR-corrected p-value (threshold p < 0.05) was calculated for each pairwise association between ROIs [34].

## Results

### Sample characteristics

Sample characteristics are shown in Table 1. Since we observed differences in age and sex, these were included as covariates in the fMRI analysis.

### rsFC alterations as a function of anxiety phenotype

#### Categorical analysis using primary diagnosis

The combined patient group exhibited increased positive connectivity between the right thalamus and right posterior insula relative to HC.

PD/AG patients exhibited decreased positive connectivity between the right subgenual ACC and the right dmPFC as well as the right pregenual ACC and the right PAG when contrasted with HC. Conversely, there was an increase in positive connectivity throughout an extensive thalamo-cortical network, encompassing the bilateral thalamus with the left amygdala, the right hippocampus, and the right posterior insula as well as the right thalamus with the left hippocampus and left posterior insula.

Compared to HC, SAD patients exhibited a decrease in negative connectivity between the right OFC and the left posterior insula.

**Fig. 2:**
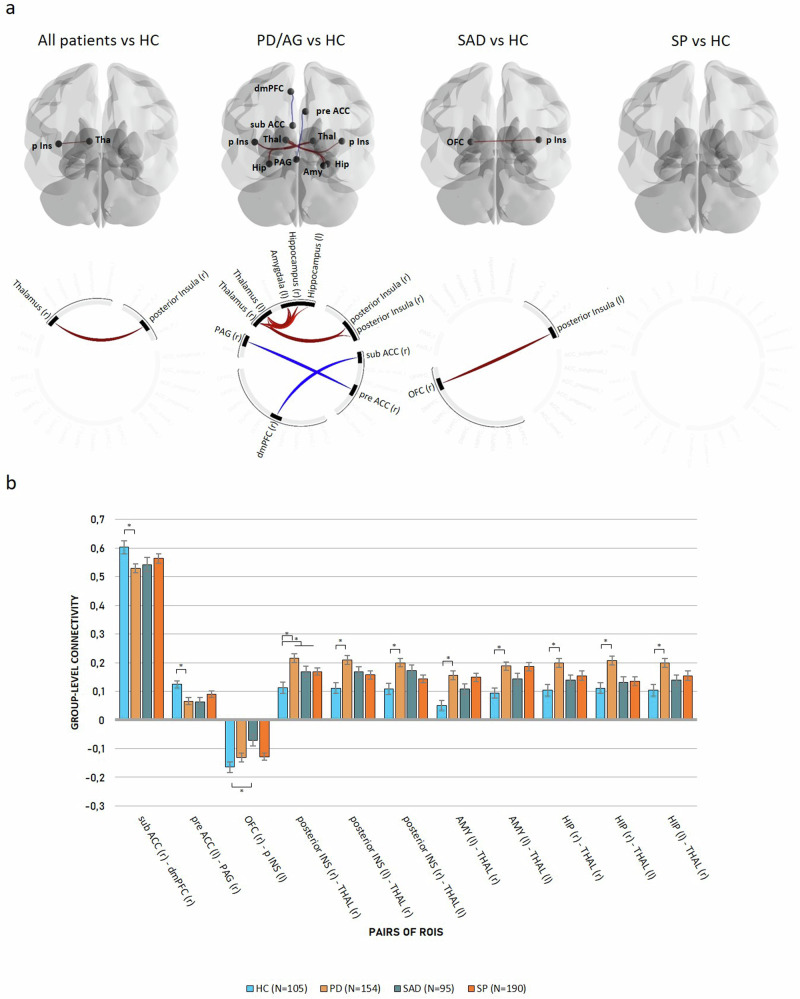
Differences in connectivity as a function of primary diagnosis. **a** Glass brain with increased connectivity in red and decreased connectivity in blue compared to HC and connectome rings with increased connectivity in red and decreased connectivity in blue compared to HC. **b** Functional connectivity values. FISHERS-Z transformed correlations as connectivity measures. * for significant connection-level FDR-corrected differences compared to healthy controls. (r) = right hemisphere; (l) = left hemisphere; p INS posterior insula; THAL Thalamus; pre ACC pregenual anterior cingulate cortex; AMY amygdala; HIP hippocampus; PAG periaqueductal gray; dmPFC dorsolateral medial prefrontal cortex; sub ACC subgenual anterior cingulate cortex; OFC orbitofrontal cortex.

SP patients displayed no discernible differences when compared to HC, as detailed in Table 2 and illustrated in Fig. 2a and b.

**Table 2:** Differences in Connectivity (ANCOVA).

Connection	Patients (m[sd])		Healthy controls		*F* (df1,df2)	*η*^*2*^	*p*
	*Z*	*SE*	*Z*	*SE*			
All patients vs. HC							
posterior Insula (l)—Thalamus (r)	0.18	0.02	0.11	0.02	15.44 (1,533)	0.03	.037
PD/AG vs HC							
pregenual ACC (l)—PAG (r)	0.07	0.01	0.12	0.01	−12.32 (1,248)	−0.05	.024
subgenual ACC (r)—dmPFC (r)	0.53	0.02	0.60	0.02	−10.89 (1,248)	−0.05	.043
posterior Insula (r)—Thalamus (r)	0.22	0.02	0.11	0.02	18.84 (1,248)	0.07	0.008
posterior Insula (r)—Thalamus (l)	0.20	0.01	0.11	0.02	14.67 (1,248)	0.06	0.009
posterior Insula(l)—Thalamus (r)	0.21	0.02	0.11	0.02	15.60 (1,248)	0.06	0.008
Hippocampus (r)—Thalamus (r)	0.20	0.02	0.10	0.02	16.24 (1,248)	0.06	0.008
Hippocampus (r)—Thalamus (l)	0.21	0.02	0.11	0.02	14.59 (1,248)	0.06	0.009
Hippocampus (l)—Thalamus (r)	0.20	0.02	0.10	0.02	12.18 (1,248)	0.05	0.024
Amygdala (l)—Thalamus (l)	0.19	0.01	0.09	0.02	16.06 (1,248)	0.06	0.008
Amygdala (l)—Thalamus (r)	0.16	0.02	0.05	0.02	17.39 (1,248)	0.07	0.008
SAD vs. HC							
posterior Insula (l)—OFC (r)	−0.07	0.02	−0.16	0.02	18.83 (1,189)	0.09	0.009
SP vs. HC							
No significant findings							

Pairs of ROIs that differed between diagnostic groups. Z—Fishers-Z transformed correlation, SE—standard error, F—F-statistic, *η*^*2*^—effect size, *p*—significance level, (r) = right hemisphere, (l) = left hemisphere.

#### Categorical analysis using any diagnosis

When compared with the categorical model focusing on the primary diagnosis, there were subtle differences in the results. For PD/AG patients, there was not a decrease in positive connectivity between the right subgenual ACC and right dmPFC. Instead, there was an increase in connectivity between the left thalamus, left posterior insula, and left hippocampus. Findings for SAD and SP remained consistent, as detailed in Table S2 of the Supplemental Material.

#### Dimensional analysis on symptom severity

There was no significant correlation between symptom severity and rsFC in the patient groups. For correlations within the patient groups between significant connectivities in the categorical model and disorder-specific symptom severity, please refer to the Supplemental Material Table S3.

#### Dimensional analysis on maximum state anxiety during scanning

PD/AD patients exhibited heightened state anxiety during scanning (refer to Fig. S2 in the Supplemental Material). To distinguish between state anxiety and psychopathology, we assessed the effects of maximum state anxiety in regions impacted by diagnosis for each patient group. We did not identify any significant associations that could account for the diagnosis-specific observations (further details available in the Supplemental Material).

## Discussion

Following the contemporary transdiagnostic and dimensional approach in research-oriented classification systems, we studied rsFC differences between HC and patients with PD/AG, SAD, and SP, focusing on both diagnostic categories and symptom severity dimensions. Our key findings are: (a) All patients compared to HCs showed increased positive connectivity between the right thalamus and the right posterior insula. Among patient groups, PD/AG patients showed the most distinct connectivity changes, especially increased connectivity between thalamo-limbic and cortical regions and decreased connectivity between the ACC and prefrontal regions and the PAG, whereas SAD patients only show changes in OFC and insula connectivity and SP patients did not show any changes; (b) these alterations were primarily associated with the categorical diagnosis and disappeared in dimensional models of symptom severity; (c) while PD/AG patients exhibited heightened state anxiety during scans, this did not account for the primary diagnosis variations. Observations suggest unique patterns for each AD group, diverging from prevalent transdiagnostic models.

The main finding across all disorders vs. HC demonstrated increased positive connectivity between the right thalamus and the right posterior insula. The thalamus serves as a critical relay station for sensory information, while the insula is heavily involved in interoceptive awareness—the processing of internal bodily states [35, 36]. The increased connectivity between these regions may suggest a heightened integration of sensory and interoceptive signals, which could contribute to the increased emotional and autonomic responses observed in anxiety disorders [37]. This connection could be indicative of enhanced interoceptive processing, potentially leading to increased awareness of bodily sensations and, consequently, autonomic symptoms such as heightened heart rate and sweating. These autonomic symptoms are commonly reported in various anxiety disorders, suggesting that this connectivity pattern might underlie a shared neurobiological mechanism contributing to the symptomatology across different diagnostic categories.

Regarding categorical differences between different ADs, only the PD/AG group exhibited notable rsFC differences compared to healthy controls. The functional anatomy of PD reveals an extensive subcortical and cortical network related to defensive reactions, highlighting areas such as the amygdala, hippocampus, thalamus, PAG, and locus coeruleus [38, 39]. Dysfunctional coordination between cortical (upstream) and brainstem (downstream) regions is believed to contribute to panic attacks [38]. PD/AG patients exhibit altered top-down and bottom-up processing dynamics in fear conditioning [40]. Both animal and human research emphasizes the significance of midbrain areas, particularly the PAG, in defensive reactions and PD [41]. Our study supports this view, showing connectivity changes in this network, including areas like the PAG, thalamus, amygdala, hippocampus, insula, ACC, and dmPFC. While we observed increased positive connectivity in limbic areas, there was a decrease between the ACC and dmPFC, as well as the ACC and PAG, reinforcing the idea of disrupted upstream and downstream processing. Although PD/AG patients reported higher anxiety during scans, this did not account for the observed results. Furthermore, no clear correlation emerged between these network changes and symptom severity, aligning with prior rsfMRI results [42]. However, earlier studies might have been less definitive due to limited sample sizes (all studies n < 55 per group; [5]). Our findings can be interpreted within a framework where defensive responses vary from prefrontal mechanisms, addressing potential threats, to midbrain mechanisms addressing immediate ones [33]. Both are vital in grasping PD’s etiopathogenesis [43, 44].

Regarding SAD specific alterations in intrinsic connectivity, only the OFC showed impaired connectivity with the insula in SAD patients in our study. Previous research on Neurofunctional models for SAD, primarily derived from task-fMRI studies, indicates alterations in fronto-limbic circuits with limbic hyperactivity and reduced activity in cognitive control areas [45]. There is also heightened activation in medial parietal and occipital regions, crucial for discerning social cues [31, 45]. Alterations in connectivity are also seen in fronto-amygdala, fronto-parietal, and amygdala-temporal networks [11], with frontal regions showing the most robust results. As such, present findings align with this body of research.

Clinically, SAD is more strongly associated with cognitive symptoms than autonomic symptoms in PD/AG. Symptoms like anxiety from social evaluation and post-social interaction processing are pivotal in sustaining SAD, often leading to rumination and preoccupation. The OFC plays a role in the higher-order assessment of emotional and social signals. Mao et al. [46] highlighted the mediating role of OFC-amygdala connectivity between social anxiety and attention biases towards emotional faces [46]. In line with our earlier research, where SAD comorbidity in PD/AG altered fear conditioning and extinction patterns [47], our latest rsfMRI findings further suggest unique neural markers for these disorders.

Despite numerous task-based fMRI studies on SP [10, 48, 49], we found only one study focusing on rsFC in such a patient group [50]. Given the null outcomes in a robustly sampled study, this disparity might point to a notable publication bias in this field. As for the absent rsFC findings in SP, it is plausible that this patient cohort has milder impairment, aligning more with the HC phenotype than PD/AG patients do, leading to differing results when compared to PD/AG. Subtle brain morphologic differences have however recently been described in an ENIGMA meta-analysis in SP [51].

Current research on disorder classification [4] focuses on identifying commonalities between disorders, thus seeking transdiagnostic explanations for psychopathology and unified treatment protocols [52]. Present findings reveal notable differences between healthy controls and PD/AG patients, some distinctiveness for SAD patients, and no differences for those with SP (direct comparisons between patient groups also show differences, see Supplemental Material). As thus, present results challenge dimensional approaches to psychopathology to a certain extent. However, more comprehensive research methods like meta- or mega-analyses, especially from vast neuroimaging consortiums like ENIGMA (https://enigma.ini.usc.edu/), which aggregates previously unreported data, should be explored for a deeper understanding of this issue [51].

While our study provides valuable insights into the rsFC differences between patient groups and healthy controls, it is important to further discuss the results of the dimensional analyses testing for associations between symptom severity and connectivity within the patient groups. Despite expectations that symptom severity would correlate with specific patterns of connectivity alterations, findings did not yield statistically significant results in this regard. This null finding challenges the assumption that rsFC alterations directly correspond to symptom severity levels within each anxiety disorder group.

Several factors may contribute to this lack of association. Firstly, it is possible that the dimensional analysis approach utilized in our study was not sensitive enough to detect subtle variations in connectivity associated with different levels of symptom severity. Our sample consists of clinical anxiety disorders and healthy controls and therefore has a bimodal distribution. This is also the reason why we investigated correlations only within the patient groups, but not across patients and controls which might have yielded spurious associations due to the bimodal distribution. Collecting a sample of healthy, subclinical, and clinically significant presentations of anxiety might help to investigate the full range of dimensional anxiety. Alternatively, the complexity of anxiety disorders, characterized by heterogeneity in symptom presentation and underlying neurobiological mechanisms, may obscure direct relationships between symptom severity and rsFC.

### Strength and limitations

Limitations encompass comorbidity, ongoing psychotropic medication, and excluded diagnostic groups related to ADs. We accounted for comorbidity by including the presence of any AD diagnosis in our analyses. Incorporating disorders like generalized anxiety disorder, obsessive-compulsive disorder, or posttraumatic stress disorder would offer a wider transdiagnostic view. Our focus was on ADs and healthy controls, but direct inter-disorder comparisons would further highlight specific signatures (see Supplemental Material). ROI selection prioritized functional associations over empirical anatomical distinctions. Using the latter would have provided a more detailed perspective with smaller ROIs. However, we believe this approach would have complicated clinical interpretation.

Despite these challenges, the absence of significant associations between symptom severity and rsFC highlights the need for further research to elucidate the complex interplay between neural connectivity patterns and different degrees of severity along a dimensional spectrum of anxiety. Future studies employing more refined analytical techniques, larger sample sizes, longitudinal designs, and epidemiological or enriched samples to reflect the full spectrum of anxiety severity (from low levels to subclinical to clinical manifestations) may provide a clearer understanding of the relationship between symptom severity and connectivity alterations, ultimately informing more targeted approaches to diagnosis and treatment.

## Conclusion

Contemporary research-oriented classifications favor a transdiagnostic and dimensional approach to mental disorders, including AD [4]. We investigated neural signatures of intrinsic functional connectivity in various ADs using rsfMRI to identify and compare differences to HCs. Findings revealed distinct rsFC changes among disorders, notably in PD/AG and SAD patients, challenging the assumption of broadly shared common factors. Pooling different AD diagnoses might overshadow information for specific disease models. More studies are needed to validate rsFC as a marker for classification and a theranostic marker for neuroplastic changes and response to treatment, paving the way for personalized, neuroscience-informed treatments.

Our results underscore categorical differences between various anxiety disorders. On one hand, this challenges transdiagnostic considerations. On the other hand, the results can be interpreted as complementary. Continuing to adopt a transdiagnostic perspective regarding mental disorders in general does not preclude individual differences that accumulate within groups. From a practical view, the state-of-the-art treatment involves exposure therapy, which is tailored to the respective anxiety disorder and its fearful expectancies. Even in broader evidence-based transdiagnostic approaches [53] of CBT, a clinician cannot avoid addressing the specific focus of anxiety which consequently includes disorder-specific components to a certain extent.

## Supplementary information

Supplement

## Data Availability

The data supporting the findings of this study are available upon reasonable request from the corresponding author. Due to the sensitive nature of the data, which includes patient health information, access is restricted to ensure compliance with the General Data Protection Regulation (GDPR) of the European Union (EU). Requests for data access will be reviewed and granted only under strict conditions that protect participant privacy and adhere to all relevant ethical and legal requirements.
