# Supplementary material for: Resting-state functional connectivity in anxiety disorders: a multicenter fMRI study
Source: Mol Psychiatry. 2024 Oct 4;30(4):1548–57. doi: 10.1038/s41380-024-02768-2 (PMC11919712; doi:10.1038/s41380-024-02768-2)
Supplement: Supplementary file 1 — Supplement [file 41380_2024_2768_MOESM1_ESM.docx]

Supplemental material

**2. Methods**

*2.1. Clinical trials and sample characteristics*

The trial of the national research consortium “Providing Tools for Effective Care and Treatment of Anxiety Disorders” (PROTECT‐AD) enrolled patients with a primary diagnosis of PD/AG, SAD or SP at eight university outpatient clinics throughout Germany. Using a multicenter randomized controlled trial accompanied by psycho-physiological, neural and (epi-)genetic add-on projects, the consortium aims to test for the critical role of extinction learning during exposure-based CBT (NIMH Protocol Registration System: 01EE1402A and German Register of Clinical Studies: DRKS00008743).

The add-on project “Neural response and fear circuitry related to extinction learning and outcome”, funded by the German Federal Ministry of Education and Research (BMBF) aimed to investigate neuro-functional differences between patients with a primary anxiety disorder and healthy controls.

The SpiderVR trial funded by the German Research Foundation was part of the Collaborative Research Center “Fear, Anxiety, Anxiety Disorders” (CRC TRR 58, project C09). It was conducted at two of the sites which also participated in PROTECT-AD (Würzburg, Münster) and who completed an identical neuroimaging backbone including rsfMRI. The study aimed to identify biobehavioral markers of treatment outcome towards *in virtuo* exposure therapy (ClinicalTrials.gov: NCT03208400).

Patient-based inclusion criteria for PROTECT-AD were 1) outpatient status, 2) age: 15–70 years, 3) current primary diagnosis of the reported anxiety disorders (panic disorder and/or agoraphobia, social anxiety disorder, and multiple specific phobia), 4) baseline severity of more than 18 points on the Hamilton Anxiety Rating Scale (SIGH-A; 28) and more than 3 points on the Clinical Global Impression scale (CGI; 29), 5) written informed consent, 6) ability to attend cognitive-behavior therapy sessions, and 7) German language competence. Exclusion criteria were 1) any current DSM-5 psychotic or substance use disorder (except nicotine), 2) concomitant psychological or psychiatric treatment (psychopharmacological medication was allowed, if dosage had been stable for at least 3 months and the medication was considered appropriate), 3) acute suicidality, and 4) general medical and fMRI contraindications (such as neurological, cardiovascular, or autoimmune diseases, pregnancy, epilepsy, or any magnetic objects on or in the body). The study protocol allowed to include patients with multiple comorbid conditions typical for routine care (such as major depression). Pilot patients were included; we did however lower some inclusion criteria such as 1) having a baseline severity of either more than 18 points on the SIGH-A OR more than 3 points on the CGI, not both. All n = 21 pilot patients in this analysis reached more than 3 points on the CGI, whereas n = 6 pilot patients did not reach 18 points on the SIGH-A. We included n = 4 pilot patients who 2) only exhibited a monosymptomatic form of specific phobia.

Inclusion criteria for SpiderVR were 1) current primary diagnosis of specific phobia animal subtype, 2) a total score above 19 in the spider phobia questionnaire (SPQ, 30), 3) age between 18 and 65 years, 4) right-handedness, 5) fluent German language skills, 6) Caucasian descent, and 7) willingness to participate in virtual-reality based exposure therapy (VRET). Patients were excluded if they fulfilled criteria for a 1) lifetime diagnosis of other comorbid anxiety disorders, obsessive-compulsive disorder, posttraumatic stress disorder, severe major depression, borderline personality disorder, bipolar I disorder, psychotic disorders, substance dependence (except nicotine), or 2) acute suicidality. Comorbid mild to moderate depression and other SP of the animal subtype were allowed if spider phobia was the primary diagnosis. Patients with 3) current psychopharmacological treatment, current or 4) past psychotherapy (including exposure), 5) neurological diseases, 6) pregnant women, and those fulfilling 7) MRI-related exclusion criteria were excluded.

*2.2 Neuroimaging data acquisition*

In both trials, several measures to guarantee a high level of data quality during the fMRI assessments were implemented.

The sequences and the scanner comparability were further evaluated before the start and across the entire data-acquisition phase by applying the same measurement sequences to MRI phantoms. A detailed study protocol was developed to obtain a homogeneous data sample. The data-acquisition was further monitored and supervised by monthly telephone conferences and site visits. In addition to the rsfMRI scan, both studies encompassed other, study specific fMRI tasks which are not reported here (both trials: emotional face recognition task (“Hariri-Task” 31), Protect-AD fear conditioning and extinction task (32); SpiderVR: phasic and sustained fear task (33).

*2.3 rsfMRI preprocessing and analysis pipeline*

In order to adjust for head motion between the slices, each participant’s movement throughout the time series was estimated and corrected for. Therefore, all functional volumes were realigned to the first scan of the timeseries (reference image) for each individual. During the realignment procedure, 6 movement parameters consisting of 3 parameters for translational head motion and 3 parameters for rotational head motion were derived for later use in nuisance regression (see below).

Due to the sequential nature of the fMRI time series, the respective slices acquired in each scan were acquired at slightly different timepoints. To account for the temporal offset, the SPM12 slice-timing correction (STC) method (34) was applied. Thus, by using sinc-interpolation, all the functional data has been shifted and resampled to adjust to the scan in the middle of each total acquisition time.

In order to elucidate the tissue organization of each individual’s brain and to map data of all participants on a standardized template for inter-subject analyses, tissue segmentation of structural data and spatial normalization of structural and functional data into MNI standard space was performed. Following the CONN *defaultMNI* pipeline, the *direct normalization* was carried out which corresponds to the SPM12 unified segmentation and normalization procedure (35). For structural segmentation and normalization, the raw T1-weighted scan was taken as a reference image whereas for functional normalization an average image of the BOLD signal was used as reference.

Tissue classification into gray matter, white matter and CSF was done by determining the tissue class probability of the voxels.

Besides segmenting the voxels into white matter, gray matter and CSF based on posterior tissue probability maps, the standard organization of voxels belonging to these different tissue classes should be considered during segmentation and registration procedures. Prior tissue probability maps show the organization of tissue classes mapped on a standard space (here: Montreal *Neurological Institute Atlas*). Based on this template, prior tissue probability maps depict the prior likelihood of voxels to pertain to a specific tissue class.

During normalization, non-linear spatial transformations of all subjects’ structural and functional data were performed to match posterior and prior tissue probability maps, until convergence. Structural and functional data were resampled to a default 180x216x180mm bounding box with 2mm isotropic voxels for functional data and 1mm for structural data.

Moreover, the unified segmentation approach (35) accounts for a problem of mutual information need of both the step of tissue class segmentation and spatial normalization. Firstly, tissue classification is based on the individual’s unstandardized brain anatomy. However, tissue probability maps that are used during the tissue classification steps follow the standard template of the MNI. Therefore, it could be argued to simply perform normalization to MNI space before tissue classification is carried out. However, information obtained through the tissue classification should be integrated for the segmentation procedure in order to align the individual’s subject’s tissues on the standard template. The unified model integrates both spatial normalization and tissue class segmentation in the same model.

Since resting-state data is particularly susceptible to participants’ movement in the scanner, the Artifact detection toolbox (ART, [www.nitrc.org/projects/artifact_detect](http://www.nitrc.org/projects/artifact_detect)) was used to identify outlier scans. An image was categorized as invalid if framewise head displacement of a respective scan exceeded 0.5 mm or if the observed global mean BOLD signal of a single scan differed more than 3 standard deviations from the total global mean BOLD signal for the entire acquisition. Patients were excluded from the sample if the number of invalid scans exceeded 25% of total scans (here ≥ 59 scans). Hence, the data of 29 patients (8,84 % of the total sample) was removed from further analysis. For each scan of patients with less than 25% outliers a first level covariate was entered for the first level analysis. This way, following the scrubbing approach, any bias of these outlier scans on the global BOLD signal was removed.

Subsequently, spatial smoothing was performed to increase the BOLD signal-to-noise ratio and to attenuate the effect of residual variability in order to increase validity of group statistics. For this purpose, the data was convolved with a 8mm full width half maximum (FWHM) Gaussian filter.

In the denoising step, remaining noise stemming from movements, physiological effects and the resting condition was removed. The 6 movement parameters from the realignment step (3 for rotational and 3 for translational motion) as well as their first-order temporal derivatives were entered into the model as first-level covariates. Moreover, nuisance regressors were generated for the effect of rest, the white matter and CSF BOLD timeseries. Based on the outlier detection step with ART, regressors for each patient with invalid scans were entered into the model for scrubbing.

After linear regression, a temporal bandpass-filter (bounding box: 0.08 Hz – 0.09 Hz) was applied on the BOLD timeseries to remove physiological artifacts (such as breathing and heartbeat) and residual movement effects of the respective participant.

*1.3. fMRI quality control*

To ensure that all data has been pre-processed correctly, quality checks as suggested by the authors of CONN were performed in CONN at several steps of the procedure.

In a first step, T1 images were visually inspected for visible artifacts throughout the data collection phase for the early detection of systematic artifacts.

The realignment step was controlled by using the QA_maxmotion viewer. The first and last acquisition from several participants were compared. No remarkable difference should be detectable between the respective scans. Additionally, second-level covariates were generated at this point to account for maximum framewise head displacement.

In order to specifically check the T2 data for any large artifacts (outlier detection), the QA_artifacts viewer was inspected. Here, a movie of the functional scans of a timeseries was viewed and global signal scan-to-scan changes and subject motion scan-to-scan displacement was visually observed. It was checked whether there were remaining artifacts that had not been detected by the ART toolbox earlier. At this point, the number of all valid scans (meaning all scans except outliers) was modeled as a second-level covariate.

A visual check of structural and functional normalization was performed using the QA_Norm viewer. The MNI gray matter boundaries were mapped on the functional data to inspect whether the match was accurate enough. No large anatomical difference should be remarkable between the respective participants. To further control for correct registration to the MNI standard, the full template, comprising gray matter, white matter and CSF ROIs were mapped on the ROIs of the functional data. White matter and CSF areas should not overlap the gray matter areas of the MNI template.

Lastly, denoising was checked in order to examine whether there are any remaining artifacts in the data. Using the QA_denoising histograms, correlations between pairs of voxels are depicted both prior to and after denoising. Since spurious correlation based on confound regressors such as motion is removed during the denoising procedure, the correlations after this step should be lower and closer centered to zero than before denoising.

In a similar manner, the QA_denoising carpet plot was inspected which depicts the BOLD signal timeseries before and after denoising. After denoising, large differences in BOLD signal between neighboring voxels (that may stem from motion or physiological noise) should visibly be smoothed out.

*1.4. ROI definition*

ROIs were generated using the brainnetome (36) atlas and the ATAG (37) atlas. The following integration of the original brainnetome regions into the ROIs were used in this study:

- dmPFC
  - label ID left: 1,9,11,13
  - label ID right: 2,10,12,14
- dlPFC
  - label ID left: 3,5,7,15,19,21,23,25
  - label ID right: 4,6,8,16,20,22,24,26,30
- OFC
  - label ID left: 27,43,45,47
  - label ID right: 28,44,46,48
- vmPFC
  - label ID left: 41,49
  - label ID right: 42,50
- vlPFC
  - label ID left: 33,35,39,51
  - label ID right: 34,35,40,52
- posterior Insula
  - label ID left: 163,169,171
  - label ID right: 164,170,172
- anterior Insula
  - label ID left: 165,167,173
  - label ID right: 166,168,174
- pregenual ACC
  - label ID left: 179
  - label ID right: 180
- dorsal ACC
  - label ID left: 183
  - label ID right: 184
- subgenual ACC
  - label ID left: 187
  - label ID right: 188
- amygdala
  - label ID left: 211,213
  - label ID right: 212,214
- hippocampus
  - label ID left: 215,217
  - label ID right: 216,218
- thalamus
  - label ID left: 231,233,235,237,239,241,243,245
  - label ID right: 232,234,236,238,240,242,244,246

For the periaqueductal gray probabilistic ROIs from the ATAG atlas were converted into fixed ROIs such that all voxels above the probability of 0.5 were used (based on masks created by 36). For further details for the prefrontal cortex see figure S1.

**Figure S1.** Subregions of prefrontal cortex (38)


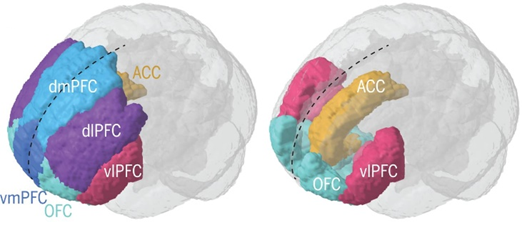


*2.5. Supplemental analyses*

For the categorical model with anxiety diagnosis either as a primary or comorbid diagnosis (“any diagnosis”) we conducted a univariate analysis of covariance comparing differences between patient groups and healthy controls. Age, sex, and scanner of fMRI acquisition were entered as covariates of no interest. Group definition led to overlapping patient groups.

For the categorical model that considered primary diagnoses, we carried out a bivariate correlation analysis between the significant connectivities and specific symptom severity. Additionally, we performed a univariate analysis of covariance to compare differences among patient groups (PD/AG vs. SAD; PD/AG vs. SP; SAD vs. SP). Age, sex, and fMRI acquisition scanner were included as covariates of no interest.

The FDR algorithm of Benjamini & Hochberg (1995) was used to control for family-wise error rates, and a connection-level FDR-corrected p-value (threshold p<0.05) was calculated for each pairwise association between ROIs.

In a post hoc analysis, we also investigated the differences in maximum anxiety (one-way ANOVA).

**3. Results**

*3.1 Categorical analysis using any diagnosis*

**Table S2. Differences in Connectivity (ANCOVA) – “any diagnosis”**

| **Connection** | **F (df1,df2)** | *η^2^* | ***p***χ 2 {\displaystyle \chi ^{2}} |
| --- | --- | --- | --- |
| **All Patients vs. HC** |  |  |  |
|  |  |  |  |
| posterior Insula (l) - Thalamus (r) | 15.44 (1,533) | 0.03 | .037 |
| **PD/AG vs. HC** |  |  |  |
| pregenual ACC (l) - PAG (r) | -11.90 (1,265) | -0.05 | .023 |
| ~~subgenual ACC (r) - dmPFC (r)~~ | ~~-10.89 (1,248)~~ | ~~-0.05~~ | ~~.043~~ |
| posterior Insula (r) - Thalamus (r) | 18.06 (1,265) | 0.06 | .004 |
| posterior Insula (r) - Thalamus (l) | 15.52 (1,265) | 0.06 | .007 |
| posterior Insula (l) - Thalamus (r) | 16.97 (1,265) | 0.06 | .004 |
| posterior *Insula (l) - Thalamus (l)* | *12.18 (1,265)* | *0.04* | *.021* |
| Hippocampus (r) - Thalamus (r) | 17.39 (1,265) | 0.06 | .004 |
| Hippocampus (r) - Thalamus (l) | 14.52 (1,265) | 0.05 | .009 |
| Hippocampus (l) - Thalamus (r) | 13.84 (1,265) | 0.05 | .012 |
| *Hippocampus (l) – Thalamus (l)* | *12.53 (1,265)* | *0.05* | *.020* |
| Amygdala (l) - Thalamus (l) | 19.71 (1,265) | 0.07 | .003 |
| Amygdala (l) - Thalamus (r) | 20.79 (1,265) | 0.07 | .003 |
| **SAD vs. HC** |  |  |  |
| posterior Insula (l) - OFC (r) | 15.45 (1,189) | 0.07 | .043 |
| **SP vs. HC** |  |  |  |
| No significant results |  |  |  |

Note. crossed out – only significant in primaryDx results; italic – only significant in anyDx results; F – F-statistic, *η^2^* – effect size, *p* – significance level, (r)=right hemisphere, (l)=left hemisphere.

*3.2 Post hoc dimensional analysis within the categorical model*

**Table S3. Correlations between connectivity and symptom severity in patient groups**

| **Connection** | ***r*** | ***p (uncorrected)*** | **Nχ 2 {\displaystyle \chi ^{2}}** |
| --- | --- | --- | --- |
| **All Patients (SIGH-A)** |  |  |  |
| posterior Insula (l) - Thalamus (r) | -.040 | .511 | 274 |
| **PD/AG (PAS)** |  |  |  |
| pregenual ACC (l) - PAG (r) | -.033 | .682 | 152 |
| subgenual ACC (r) - dmPFC (r) | -.134 | .099 | 152 |
| posterior Insula (r) - Thalamus (r) | -.047 | .563 | 152 |
| posterior Insula (r) - Thalamus (l) | .054 | .508 | 152 |
| posterior Insula(l) - Thalamus (r) | -.062 | .449 | 152 |
| Hippocampus (r) - Thalamus (r) | .117 | .152 | 152 |
| Hippocampus (r) - Thalamus (l) | .093 | .257 | 152 |
| Hippocampus (l) - Thalamus (r) | .094 | .248 | 152 |
| Amygdala (l) - Thalamus (l) | .090 | .272 | 152 |
| Amygdala (l) - Thalamus (r) | -.001 | .986 | 152 |
| **SAD (LSAS)** |  |  |  |
| posterior Insula (l) - OFC (r) | .053 | .615 | 95 |
|  |  |  |  |
| **SP** |  |  |  |
| No significant findings |  |  |  |

Note: SIGH‐A, Structured Interview Guide for the Hamilton Anxiety Rating Scale; PAS, Panic and Agoraphobia Scale; LSAS, Liebowitz Social Anxiety Scale

*3.3 Categorical analysis using primary diagnosis for direct comparisons between patient groups*

**Table S4. Differences in connectivity (ANCOVA) between patient groups**

| **Connection** | **F (df1,df2)** | *η^2^* | ***p***χ 2 {\displaystyle \chi ^{2}} |
| --- | --- | --- | --- |
| **PD/AG vs. SAD** |  |  |  |
| vmPFC (r) - dlPFC (l) | 4.04 (1,238) | 0.02 | .025 |
| dmPFC (r) - Hippocampus (r) | -3.88 (1,238) | 0.02 | .025 |
| **PD/AG vs. SP** |  |  |  |
| No significant results |  |  |  |
| **SAD vs. SP** |  |  |  |
| posterior Insula (l) - OFC (r) | 4.27 (1,274) | 0.02 | 0.01 |
| posterior Insula (r) - OFC (r) | 3.68 (1,274) | 0.01 | 0.04 |
| posterior Insula (l) - dlPFC (r) | 3.67 (1,274) | 0.01 | 0.04 |

Note. F – F-statistic, *η^2^* – effect size, *p* – significance level, (r)=right hemisphere, (l)=left hemisphere.

*3.4 Post-hoc dimensional analysis on maximum state anxiety during scanning*

A one-way ANOVA was performed to compare the effect of diagnosis on maximum anxiety. There was a statistically significant effect of diagnosis on maximum anxiety (F(3) = 48.38, p < .001).

Tukey’s HSD Test for multiple comparisons found that the mean value of maximum anxiety was significantly different between PD/AG and SAD (p = .007 95% C.I. = [.20, 1.75]), between PD/AG and HC (p < .001, 95% C.I. = [2.73, 4.23]), between SAD and HC (p < .001 95% C.I. = [1.67, 3.34]), and between SP and HC (p < .001 95% C.I. = [1.11, 3.78]). See figure S2.

**Figure S2. Maximum Anxiety during scanning Session**


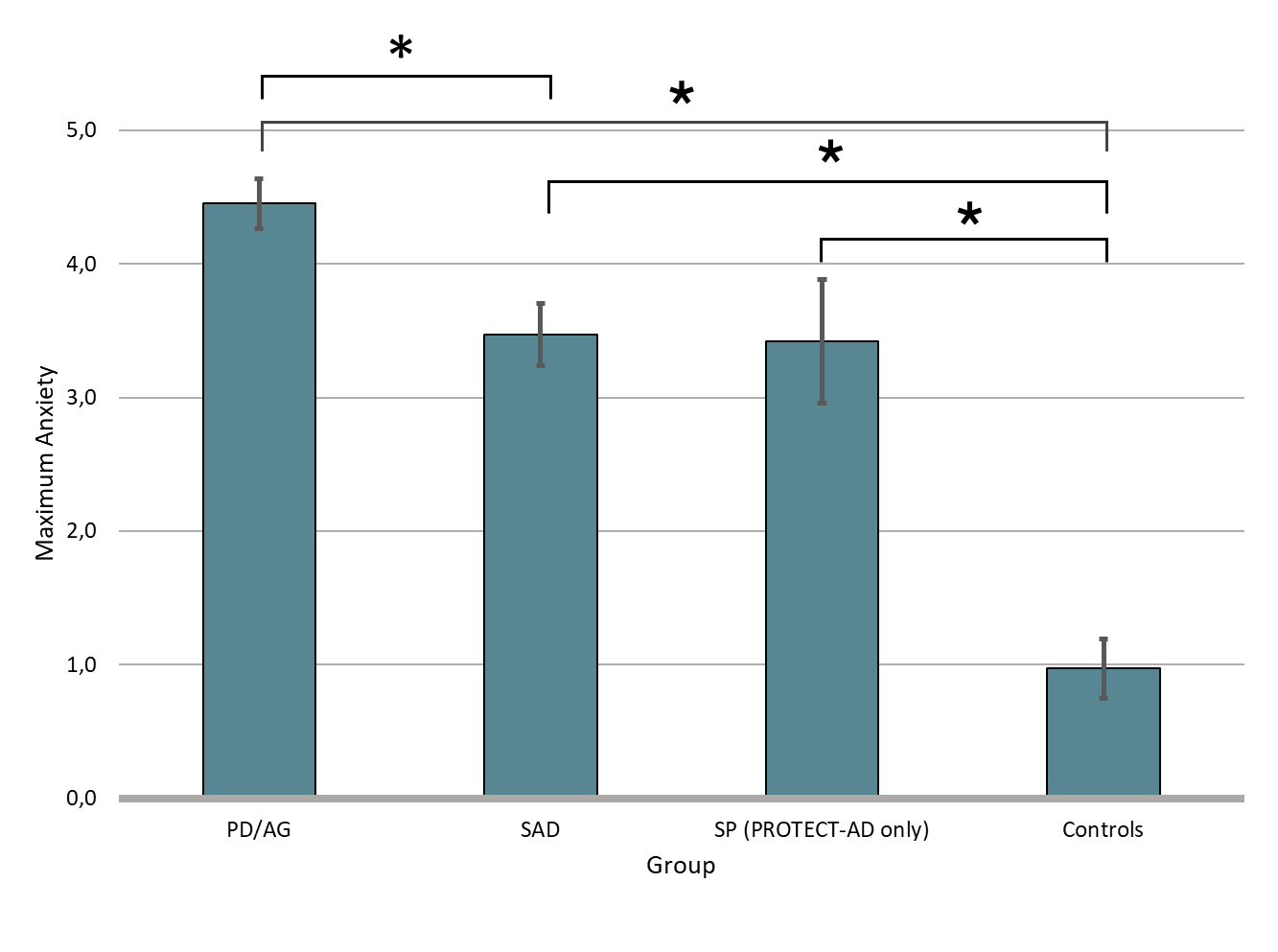


**Figure S3. Association between connectivity and maximum anxiety**

**A: All patients vs. controls**

**
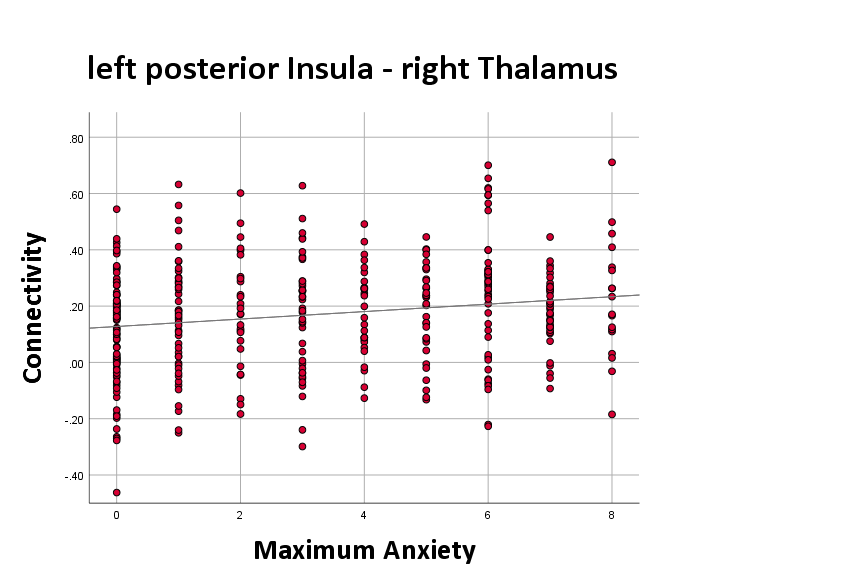
**

T = 1.54

p = .998

**B: PD/AG vs. controls**


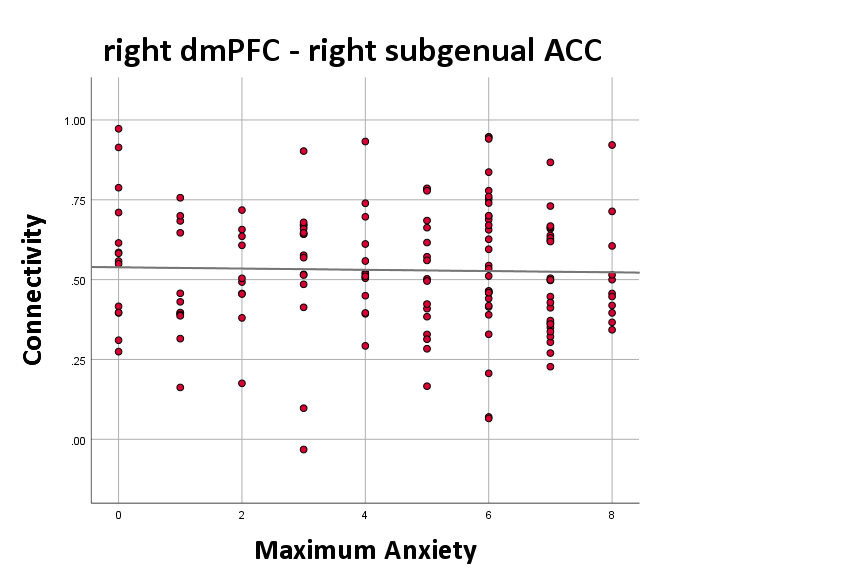

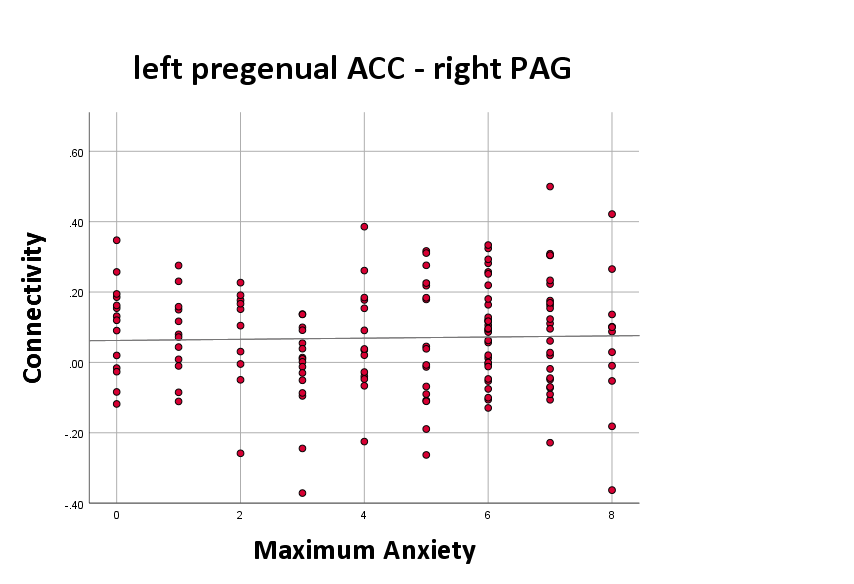

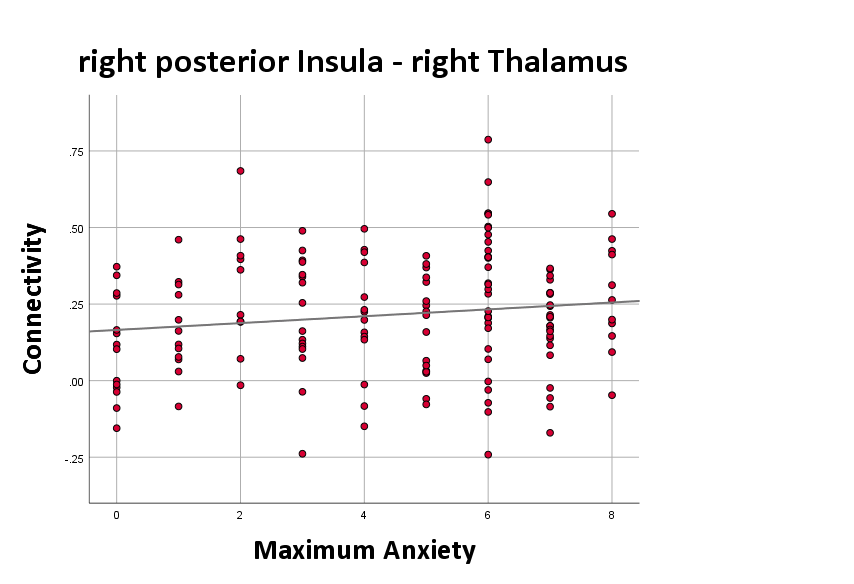

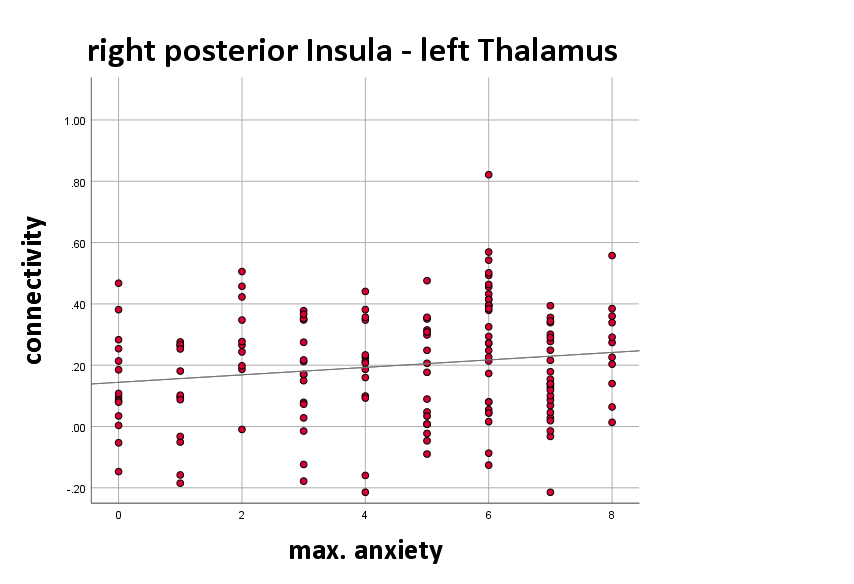

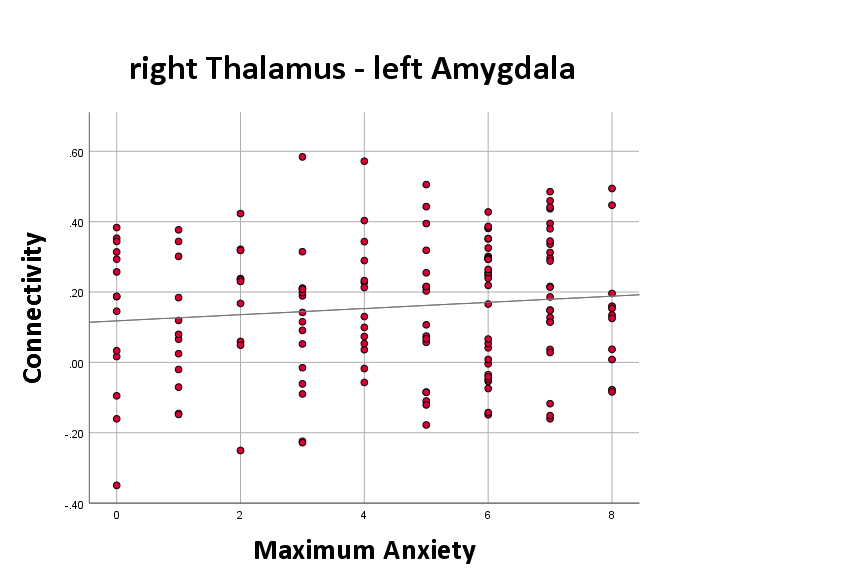

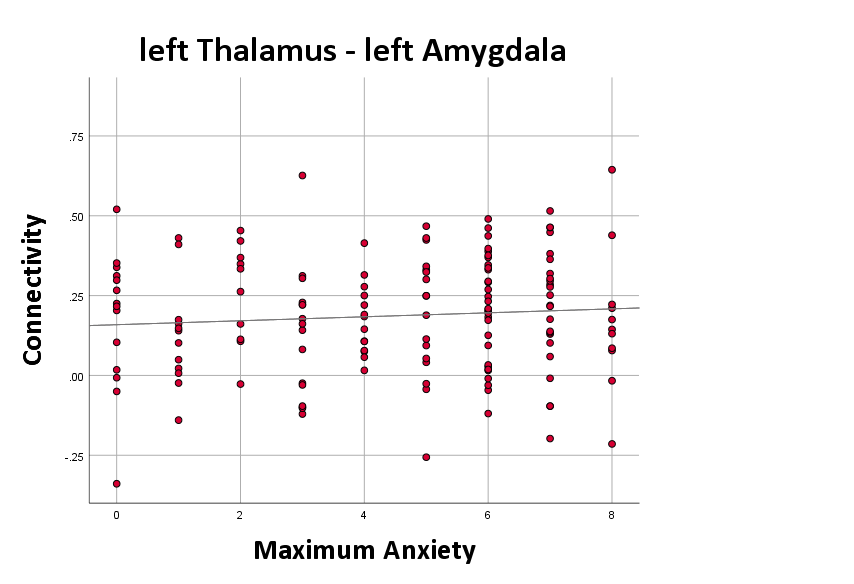

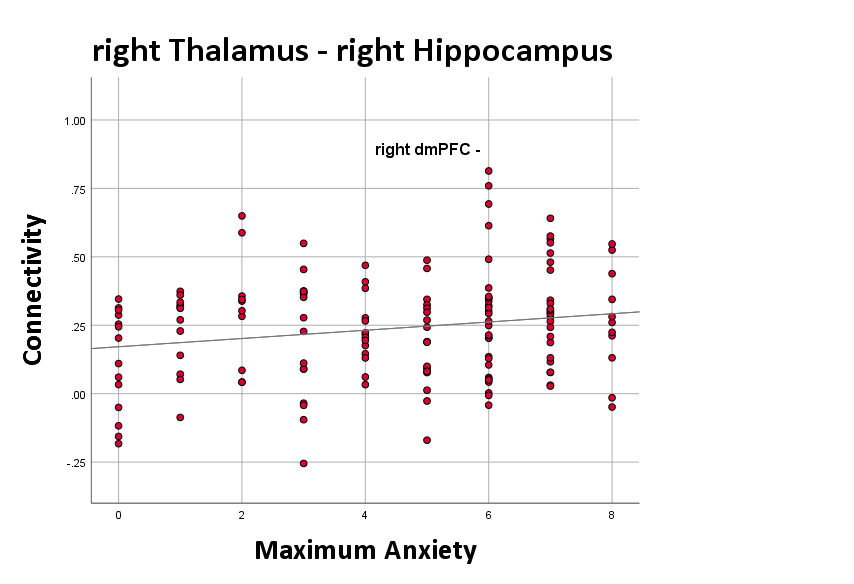

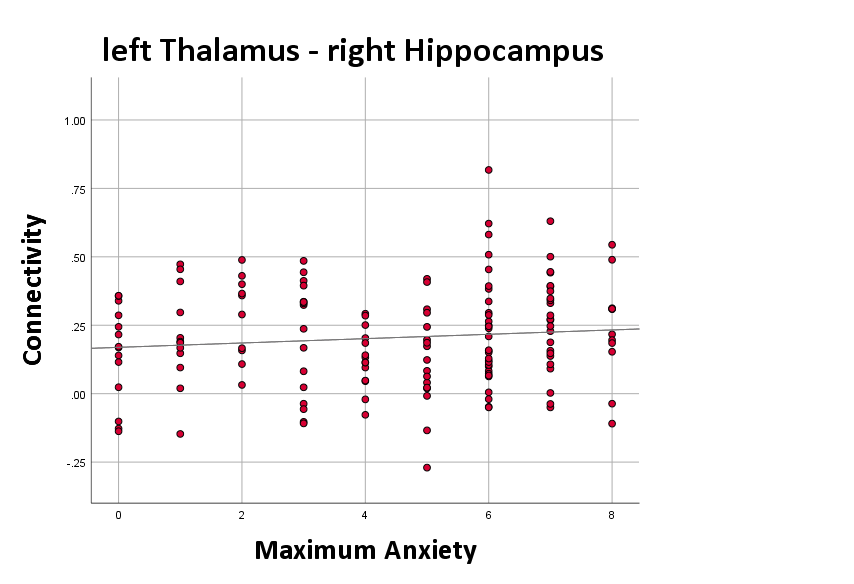


T = 1.76

p = .576

T = 1.58

p = .671

T = .83

p = .809

T = -1.83

p = .573

T = 1.42

p = .699

T = .79

p = .810

T = 2.18

p = .560

T = .97

p = .257


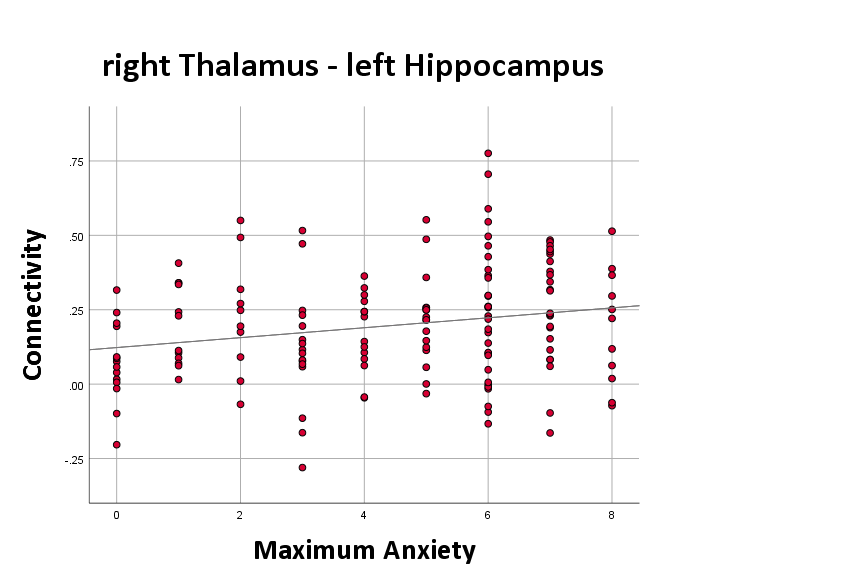


T = 2.36

p = .560

C: SAD vs. controls


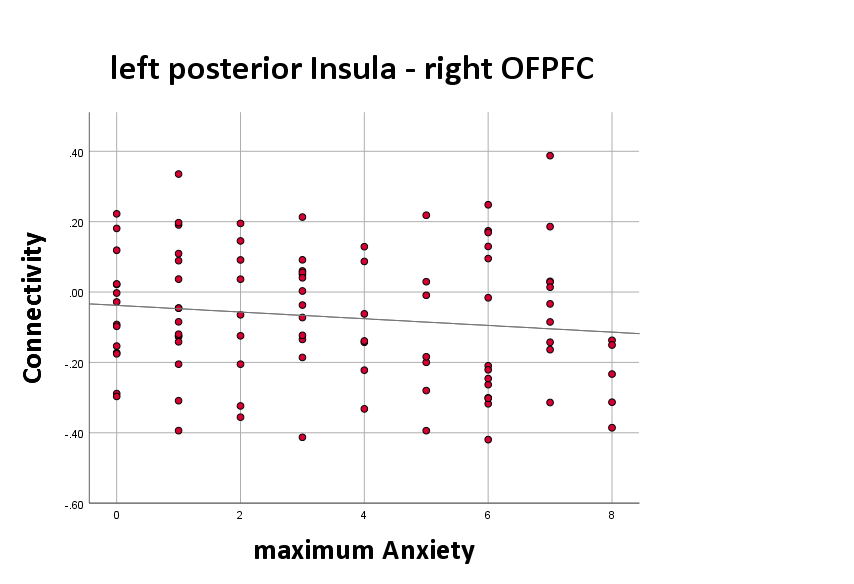


β = -.132

p = .202

Note. T – T-statistic, *p* – FDR corrected significance level

References

1. Zhu H, Qiu C, Meng Y, Yuan M, Zhang Y, Ren Z, u. a. Altered Topological Properties of Brain Networks in Social Anxiety Disorder: A Resting-state Functional MRI Study. Sci Rep. März 2017;7(1):43089.

2. Yuan M, Zhu H, Qiu C, Meng Y, Zhang Y, Ren Z, u. a. Altered regional and integrated resting-state brain activity in general social anxiety disorder patients before and after group cognitive behavior therapy. Psychiatry Research: Neuroimaging. Februar 2018;272:30–7.

3. Yuan M, Zhu H, Qiu C, Meng Y, Zhang Y, Shang J, u. a. Group cognitive behavioral therapy modulates the resting-state functional connectivity of amygdala-related network in patients with generalized social anxiety disorder. BMC Psychiatry. 13. Juni 2016;16:198.

4. Yang X, Liu J, Meng Y, Xia M, Cui Z, Wu X, u. a. Network analysis reveals disrupted functional brain circuitry in drug-naive social anxiety disorder. NeuroImage. April 2019;190:213–23.

5. Shin YW, Dzemidzic M, Jo HJ, Long Z, Medlock C, Dydak U, u. a. Increased resting-state functional connectivity between the anterior cingulate cortex and the precuneus in panic disorder: Journal of Affective Disorders. September 2013;150(3):1091–5.

6. Rabany L, Diefenbach GJ, Bragdon LB, Pittman BP, Zertuche L, Tolin DF, u. a. Resting-State Functional Connectivity in Generalized Anxiety Disorder and Social Anxiety Disorder: Evidence for a Dimensional Approach. Brain Connectivity. Juni 2017;7(5):289–98.

7. Qiu C, Liao W, Ding J, Feng Y, Zhu C, Nie X, u. a. Regional homogeneity changes in social anxiety disorder: a resting-state fMRI study. Psychiatry Res. 31. Oktober 2011;194(1):47–53.

8. Prater KE, Hosanagar A, Klumpp H, Angstadt M, Phan KL. Aberrant amygdala-frontal cortex connectivity during perception of fearful faces and at rest in generalized social anxiety disorder. Depress Anxiety. März 2013;30(3):234–41.

9. Pannekoek JN, Veer IM, van Tol MJ, van der Werff SJA, Demenescu LR, Aleman A, u. a. Aberrant limbic and salience network resting-state functional connectivity in panic disorder without comorbidity. Journal of Affective Disorders. Februar 2013;145(1):29–35.

10 .Pang M, Zhong Y, Hao Z, Xu H, Wu Y, Teng C, u. a. Resting-state causal connectivity of the bed nucleus of the stria terminalis in panic disorder. Brain Imaging and Behavior

11.Neufang S, Geiger MJ, Homola GA, Mahr M, Schiele MA, Gehrmann A, u. a. Cognitive-behavioral therapy effects on alerting network activity and effective connectivity in panic disorder. Eur Arch Psychiatry Clin Neurosci. August 2019;269(5):587–98.

12.Manning J, Reynolds G, Saygin ZM, Hofmann SG, Pollack M, Gabrieli JDE, u. a. Altered Resting-State Functional Connectivity of the Frontal-Striatal Reward System in Social Anxiety Disorder. Walter M, Herausgeber. PLoS ONE. 30. April 2015;10(4):e0125286.

13.Liu F, Zhu C, Wang Y, Guo W, Li M, Wang W, u. a. Disrupted cortical hubs in functional brain networks in social anxiety disorder. Clin Neurophysiol. September 2015;126(9):1711–6.

14.Liu F, Guo W, Fouche JP, Wang Y, Wang W, Ding J, u. a. Multivariate classification of social anxiety disorder using whole brain functional connectivity. Brain Struct Funct. Januar 2015;220(1):101–15.

15.Liao W, Qiu C, Gentili C, Walter M, Pan Z, Ding J, u. a. Altered effective connectivity network of the amygdala in social anxiety disorder: a resting-state FMRI study. PLoS One. 22. Dezember 2010;5(12):e15238.

16. Liao W, Chen H, Feng Y, Mantini D, Gentili C, Pan Z, u. a. Selective aberrant functional connectivity of resting state networks in social anxiety disorder. Neuroimage. 1. Oktober 2010;52(4):1549–58.

17.Lai CH, Wu YT. The alterations in inter-hemispheric functional coordination of patients with panic disorder: The findings in the posterior sub-network of default mode network. Journal of Affective Disorders. September 2014;166:279–84.

18.Lai CH, Wu YT. Patterns of fractional amplitude of low-frequency oscillations in occipito-striato-thalamic regions of first-episode drug-naïve panic disorder. Journal of Affective Disorders. Dezember 2012;142(1–3):180–5.

19.Klumpp H, Keutmann MK, Fitzgerald DA, Shankman SA, Phan KL. Resting state amygdala-prefrontal connectivity predicts symptom change after cognitive behavioral therapy in generalized social anxiety disorder. Biol Mood Anxiety Disord. Dezember 2014;4(1):14.

20.Jung YH, Shin JE, Lee YI, Jang JH, Jo HJ, Choi SH. Altered Amygdala Resting-State Functional Connectivity and Hemispheric Asymmetry in Patients With Social Anxiety Disorder. Front Psychiatry. 26. April 2018;9:164.

21.Hang Y, Zhong Y, Zhang G, Wu Z, Kong J, Wang Q, u. a. Altered spontaneous neural activity in frontal and visual regions in patients with acrophobia. Journal of Affective Disorders. 15. April 2022;303:340–5.

22.Hahn A, Stein P, Windischberger C, Weissenbacher A, Spindelegger C, Moser E, u. a. Reduced resting-state functional connectivity between amygdala and orbitofrontal cortex in social anxiety disorder. NeuroImage. Juni 2011;56(3):881–9.

23.Geiger MJ, Domschke K, Ipser J, Hattingh C, Baldwin DS, Lochner C, u. a. Altered executive control network resting-state connectivity in social anxiety disorder. The World Journal of Biological Psychiatry. 2. Januar 2016;17(1):47–57.

24.Fitzgerald JM, Klumpp H, Langenecker S, Phan KL. Transdiagnostic Neural Correlates of Volitional Emotion Regulation in Anxiety and Depression. Depress Anxiety. Mai 2019;36(5):453–64.

25.Dodhia S, Hosanagar A, Fitzgerald DA, Labuschagne I, Wood AG, Nathan PJ, u. a. Modulation of Resting-State Amygdala-Frontal Functional Connectivity by Oxytocin in Generalized Social Anxiety Disorder. Neuropsychopharmacol. August 2014;39(9):2061–9.

26.Ding J, Chen H, Qiu C, Liao W, Warwick JM, Duan X, u. a. Disrupted functional connectivity in social anxiety disorder: a resting-state fMRI study. Magnetic Resonance Imaging. Juni 2011;29(5):701–11.

27. Arnold Anteraper S, Triantafyllou C, Sawyer AT, Hofmann SG, Gabrieli JD, Whitfield-Gabrieli S. Hyper-Connectivity of Subcortical Resting-State Networks in Social Anxiety Disorder. Brain Connectivity. März 2014;4(2):81–90.

28.Shear MK, Vander Bilt J, Rucci P, Endicott J, Lydiard B, Otto MW, u. a. Reliability and validity of a structured interview guide for the Hamilton Anxiety Rating Scale (SIGH-A). Depress Anxiety. 2001;13(4):166–78.

29.Guy W. ECDEU Assessment Manual for Psychopharmacology. US Department of Health, Education, and Welfare, Public Health Service; 1976.

30.Hamm A. Spezifische Phobien. Göttingen: Hogrefe; 2006. (Fortschritte der Psychotherapie).

31.Hariri AR, Tessitore A, Mattay VS, Fera F, Weinberger DR. The amygdala response to emotional stimuli: a comparison of faces and scenes. NeuroImage. September 2002;17(1):317–23.

32.Ridderbusch IC, Wroblewski A, Yang Y, Richter J, Hollandt M, Hamm AO, u. a. Neural adaptation of cingulate and insular activity during delayed fear extinction: A replicable pattern across assessment sites and repeated measurements. NeuroImage. 15. August 2021;237:118157.

33.Münsterkötter AL, Notzon S, Redlich R, Grotegerd D, Dohm K, Arolt V, u. a. SPIDER OR NO SPIDER? NEURAL CORRELATES OF SUSTAINED AND PHASIC FEAR IN SPIDER PHOBIA. Depress Anxiety. September 2015;32(9):656–63.

34.Henson RNA, Buechel C, Josephs O, Friston KJ. The slice-timing problem in event-related fMRI. NeuroImage. 1999;9:125-.

35.Ashburner J, Friston KJ. Unified segmentation. Neuroimage. 2005;26(3):839–51.

36.Fan L, Li H, Zhuo J, Zhang Y, Wang J, Chen L, u. a. The Human Brainnetome Atlas: A New Brain Atlas Based on Connectional Architecture. Cereb Cortex. August 2016;26(8):3508–26.

37.Keuken MC, Bazin PL, Backhouse K, Beekhuizen S, Himmer L, Kandola A, u. a. Effects of aging on T₁, T₂*, and QSM MRI values in the subcortex. Brain Struct Funct. August 2017;222(6):2487–505.

38.Carlén M. What constitutes the prefrontal cortex? Science. 27. Oktober 2017;358(6362):478–82.
